# Beta Cells within Single Human Islets Originate from Multiple Progenitors

**DOI:** 10.1371/journal.pone.0003559

**Published:** 2008-10-29

**Authors:** Raphaël Scharfmann, Xiangwei Xiao, Harry Heimberg, Jacques Mallet, Philippe Ravassard

**Affiliations:** 1 University Paris-Descartes, Faculty of Medicine, INSERM, Necker Hospital, U845, Paris, France; 2 Diabetes Research Center, Vrije Universiteit Brussel, Brussels, Belgium; 3 Centre National de la Recherche Scientifique and Université Pierre et Marie Curie, LGN UMR 7091, Paris, France; University of Sydney, Australia

## Abstract

**Background:**

In both humans and rodents, glucose homeostasis is controlled by micro-organs called islets of Langerhans composed of beta cells, associated with other endocrine cell types. Most of our understanding of islet cell differentiation and morphogenesis is derived from rodent developmental studies. However, little is known about human islet formation. The lack of adequate experimental models has restricted the study of human pancreatic development to the histological analysis of different stages of pancreatic development. Our objective was to develop a new experimental model to (i) transfer genes into developing human pancreatic cells and (ii) validate gene transfer by defining the clonality of developing human islets.

**Methods and Findings:**

In this study, a unique model was developed combining *ex vivo* organogenesis from human fetal pancreatic tissue and cell type-specific lentivirus-mediated gene transfer. Human pancreatic progenitors were transduced with lentiviruses expressing GFP under the control of an insulin promoter and grafted to severe combined immunodeficient mice, allowing human beta cell differentiation and islet morphogenesis. By performing gene transfer at low multiplicity of infection, we created a chimeric graft with a subpopulation of human beta cells expressing GFP and found both GFP-positive and GFP-negative beta cells within single islets.

**Conclusion:**

The detection of both labeled and unlabeled beta cells in single islets demonstrates that beta cells present in a human islet are derived from multiple progenitors thus providing the first dynamic analysis of human islet formation during development. This human transgenic-like tool can be widely used to elucidate dynamic genetic processes in human tissue formation.

## Introduction

The mature pancreas consists of acinar cells that produce digestive enzymes secreted via the ducts into the intestine and hormone-producing endocrine cells. The endocrine cells are organized into micro-organs called islets of Langerhans. In the adult pancreas, the islets of Langerhans (a few thousand per pancreas in rodents, about 1 million in humans) are dispersed throughout the exocrine tissue and represent 1–2% of the total pancreatic mass. Each islet contains alpha, beta, delta, PP and epsilon cells that synthesize and release glucagon (alpha), insulin (beta), somatostatin (delta), pancreatic polypeptide (PP) and ghrelin (epsilon cells) in a nutrient-dependent fashion. Beta cells, the most abundant islet cell type, are major regulators of blood sugar levels in mammals. Although rodent islet cell development is well studied [1], [2], little is known about islet formation in humans.

In rodents, islets form during the prenatal period, from multipotent endodermal pancreatic progenitor cells; these progenitor cells express the homeodomain-containing transcription factor *Pdx1 (Pancreatic duodenal homeobox-1)*, are located in the epithelial tree [3]–[5], and are proliferative [6], [7]. Progenitor cells entering the endocrine pathway express the basic helix-loop-helix transcription factor *Neurogenin3* [8], [9]. They delaminate, migrate and aggregate to form the islets of Langerhans [10], [11]. In rodent islets, beta cells are clustered in the center of the micro-organ and surrounded by other endocrine cell types, whereas, in humans, the various endocrine cells are found scattered within the islets [12], [13]. In rodents, beta cells within single islets develop from several independent progenitors [14]. However, to the best of our knowledge, this has not been demonstrated for human islets.

In this study, we demonstrated that beta cells present in a human islet develop from multiple progenitors.

## Results

### *Ex vivo* model of human islet development

**Figure 1:**
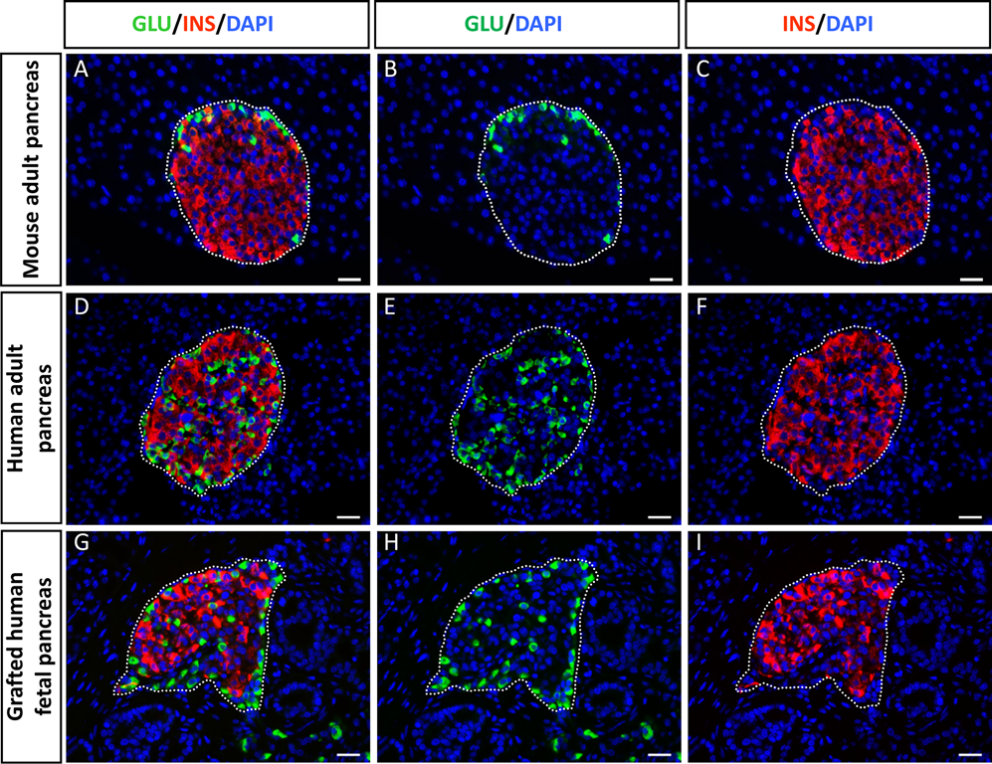
Cytoarchitecture of newly formed islets from human fetal pancreas. A–C: Section of adult mouse pancreas stained for glucagon (green) and insulin (red). D–F: Section of an adult human pancreas stained for glucagon (green) and insulin (red). G–I: Section of a human fetal pancreas analyzed 4.5 months after transplantation and stained for glucagon (green) and insulin (red). Nuclear staining (blue) was performed with DAPI. Scale bars: 25 µm.

In human fetuses, the first insulin-positive cells appear between seven and eight weeks of development; these cells go on to associate to form islets of Langerhans [17]–[19]. We have previously shown that islet formation occurs when human fetal pancreases are grafted under the kidney capsule of *scid* mice [16], [20]. In this study, we first compared the cyto-architecture of islets that developed from human fetal pancreas transplanted in *scid* mice to that of islets found in human and mouse pancreas. Adult mouse islets are composed of an insulin-positive cell core surrounded by glucagon-expressing cells (Fig. 1, Panels A–C), whereas in human adult islets (Fig. 1, panels D–F), glucagon-expressing cells are scattered throughout the islets [13], [21]. In our study, the distribution of insulin- and glucagon-positive cells within a human islet 4.5 months after transplantation was very similar to that observed for human adult pancreatic islets (Fig. 1 compare panel G–I to D–F).

**Figure 2:**
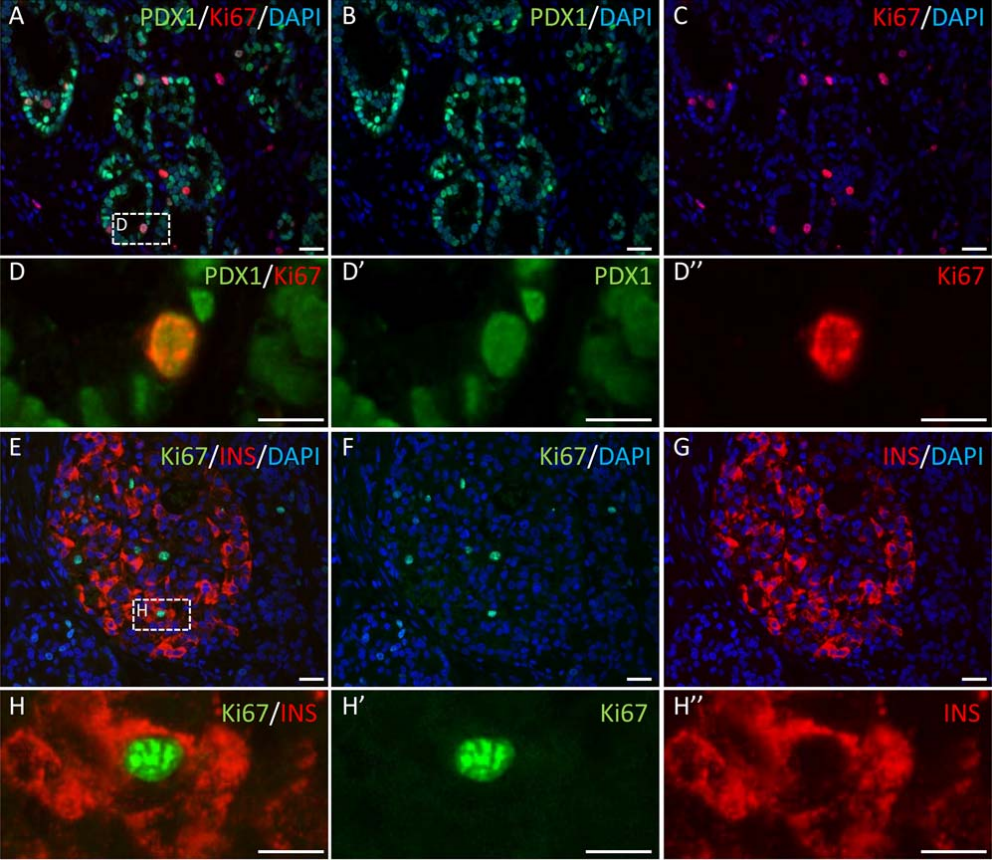
Both human pancreatic progenitors and beta cells proliferate. Human embryonic pancreases were transplanted into *scid* mice. Ten days and 4.5 months later, the grafts were removed and analyzed. A–D: Representative section of a fetal pancreas analyzed 10 days after transplantation and double stained for Pdx1 (green) and Ki67 (red). E–H: Representative section of a fetal pancreas analyzed 4.5 months after transplantation and double stained for insulin (red) and Ki67 (green). Scale bars: A–C and E–G: 25 µm; D and H: 10 µm.

During development of the islet, beta cells are formed from Pdx1-positive pancreatic progenitors located within the epithelial tree. We investigated whether Pdx1-positive cells or beta cells themselves proliferate in human pancreases transplanted into *scid* mice. At 10 days after transplantation, Pdx1-positive cells from the grafted fetal pancreas were expressing Ki67, indicating activation of the cell cycle in these cells (Fig 2A–D). Only a few insulin-positive cells were present at this stage of differentiation and these cells did not express Ki67 (not shown). Four and a half months after transplantation, the majority of beta cells were negative for Ki67, but we observed a few proliferating insulin-positive cells (Fig 2E–H).

### Human islet beta cells are derived from more than one progenitor

**Figure 3:**
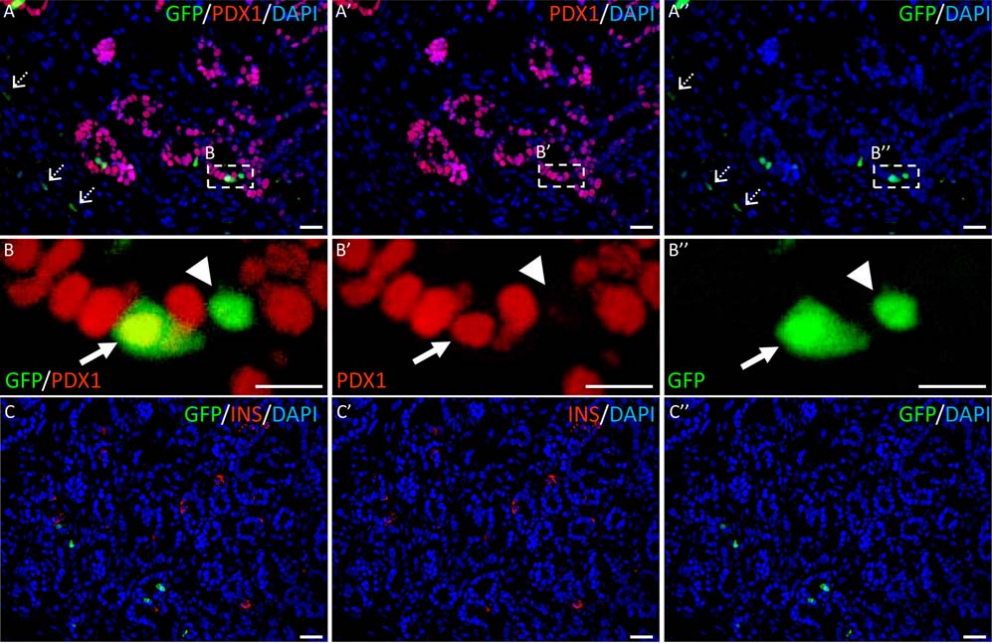
Lentivirus-mediated gene transfer in human fetal pancreas. Human fetal pancreases were partially dissociated, transduced with lentiviruses expressing GFP under the control of the CMV promoter, grafted into scid mice and analyzed 10 days later. A double staining for Pdx1 (red) and GFP (green); B represent enlargements of A. Dotted arrows: infected fibroblastic-like cells. Arrows: Pdx1-positive cells. Arrow head: Pdx1–negative cells. C double staining for insulin (red) and GFP. Scale bars: A, C: 25 µm, B: 10 µm.

To trace human fetal pancreatic progenitor cells, we first transduced partially dissociated fetal pancreases with replication-defective lentiviruses expressing eGFP under the control of the ubiquitous human cytomegalovirus (CMV) promoter, at low multiplicity of infection (MOI). Transduced pancreases were transplanted under the kidney capsule of *scid* mice. After 10 days, we observed eGFP-expressing fibroblastic-like cells (Fig 3 A, arrows). The rare scattered insulin-positive cells were found negative for eGFP (Fig. 3C). Epithelial Pdx1-positive (Fig 3 B, arrow) and Pdx1-negative (Fig 3 B, arrowhead) cells were observed in these grafts. As expected with low MOI, only 0.6% of Pdx1-positive cells expressed eGFP (123 out of 20,000 Pdx1-positive cells counted). Such low percentage enables us to determine whether human islet beta cells are derived from one or several progenitors.

**Figure 4:**
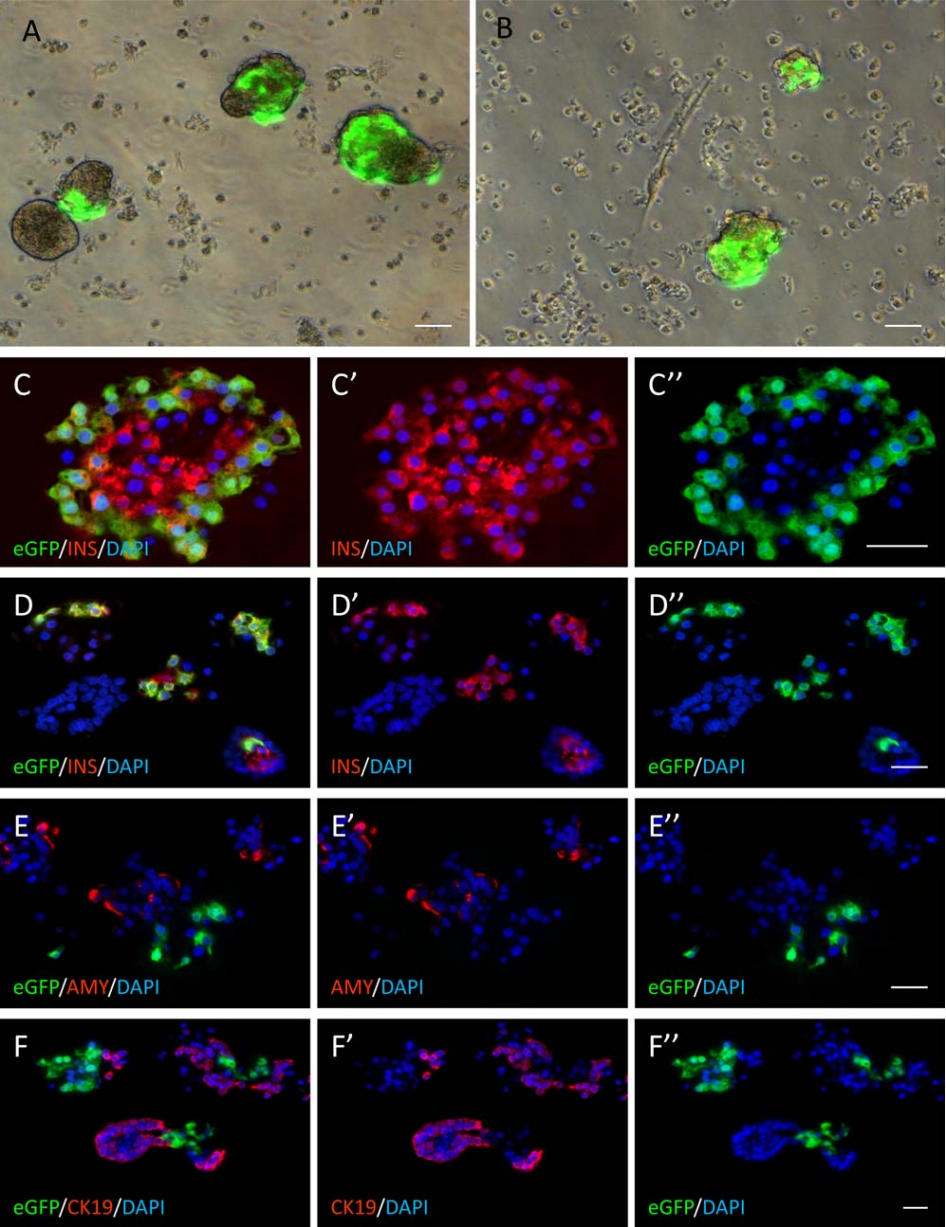
Cell type specificity of the rat insulin II promoter in human adult pancreas. Crude human islet preparations were transduced with lentiviruses expressing eGFP under the control of the rat insulin II promoter and analyzed 72 hours after infection. Cultures were photographed under a fluorescent inverted microscope (panels A and B), fixed and sectioned. C–D: staining for GFP (green) and insulin (red). E staining for GFP (green) and amylase (red). F: staining for GFP (green) and CK19 (red). Scale bars: 20 µm.

We next transduced human fetal pancreases with lentiviral vector expressing eGFP under the control of a 405 bp-fragment of the rat insulin II promoter (RIP405). This portion of the rat promoter is sufficient for beta cell restricted expression in rodents [15]. To determine whether this also applies for human cells, an islet cell-enriched fraction of pancreatic cells was transduced with pTRIP ΔU3.RIP405-eGFP. We observed intense staining in cell clusters (Fig. 4, panels A and B). We then sectioned clusters and co-stained for eGFP and endocrine, acinar or ductal markers. All eGFP-positive cells stained positive for insulin (Fig. 4, panel C, D) whereas neither amylase-expressing acinar cells, nor cytokeratin 19-expressing ductal cells (Fig. 4, panel E, F) stained positive for eGFP. Thus, this 405 bp fragment of the rat insulin II promoter is sufficient for beta cell-restricted expression of a transgene in the human adult pancreas.

**Figure 5:**
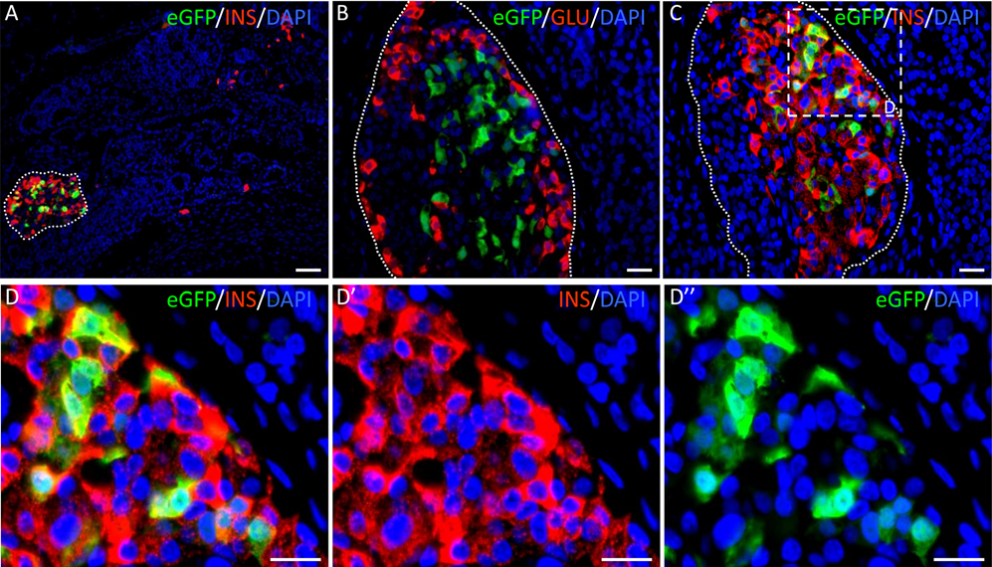
Human beta cells within a single islet are derived from more than one progenitor. Human fetal pancreases were dissociated, transduced with lentiviruses expressing GFP under the control of the insulin promoter, grafted and analyzed 4.5 months later. A: double staining for insulin (red) and GFP (green) showing that GFP is only found in islets; B: double staining for glucagon (red) and GFP (green) showing that GFP is not found in alpha cells; C: double staining for insulin (red) and GFP (green) showing that GFP is only found in a subpopulation of beta cells. D is an enlargement of panel C (dotted square). Scale bars: A: 50 µm; B–D: 25 µm.

We finally transduced human fetal pancreases with pTRIP ΔU3.RIP405-eGFP at the multiplicity of infection shown to target a limited proportion of progenitors. Pancreases were then grafted into *scid* mice and removed 4.5 months later. Islets of Langerhans had developed during the transplantation period (Fig. 5A). eGFP expression was specific to beta cells within the islets (Fig. 5, panels A, B and C), consistent with the specificity of the rat insulin II promoter. If all beta cells within an islet are derived from a single progenitor, they should all be positive for eGFP. However, if they are derived from more than one progenitor, a mixed pattern should be found. We counted 2730 islets and found that 23 contained eGFP-positive cells. In all labeled islets, only a subpopulation of insulin-positive cells was positive for eGFP (Fig. 5). These findings demonstrate that beta cells within a single human islet are derived from more than one progenitor.

## Discussion

We combined two experimental approaches — a transplantation model of human fetal pancreas that recapitulates development of human pancreatic islets and lentivirus-mediated gene transfer — to demonstrate that beta cells from a single human islet are derived from multiple progenitors.

In mice, one previous study has used chimeras to demonstrate that each islet consists of a mixture of beta cells originating from a pool of progenitor cells [14], but this has not been determined for human islets. Overall, information on pancreas development, beta-cell differentiation and islet morphogenesis in human pancreas is rare compared to rodent [23]. This is due to limited access to human tissues, as well as to lack of experimental systems to study human pancreatic development. It is currently believed that human and rodent islets develop the same way. Thus, it has been suggested that beta cells may be generated from human embryonic stem cells, based on our knowledge of rodent pancreatic development [25]. However, rodent and human islets share many similarities, but also differ substantially. For example, cell proliferation, susceptibility to destruction and beta-cell function differ between rodents and humans [26]–[29]. Moreover, islet cytoarchitecture, possibly with functional implications, also differs [13], [21].

Our knowledge of human pancreatic development is mainly based on previous studies comparing human pancreatic sections taken at various stages of development [17]–[19], [22], [30]. However, tissue sections are not informative in determining whether beta cells from a single islet develop from one or several progenitors. Nor is the aggregation chimera system used for rodents [14] applicable to address this issue in humans. In fact, few dynamic models mimicking islet cell development are available [16], [31]. Here, we developed a novel experimental system based on lentivirus-mediated gene transfer in a reconstituted model of human pancreatic development. We previously showed that development of human fetal pancreas transplanted under the kidney capsule of immuno-incompetent *scid* mice reflects that of normal human pancreas development [16], [20]. Within the graft endocrine cells differentiate and functional human islets of Langerhans containing all endocrine cell types are formed after a few months [16]. In this study, we showed that the cytoarchitecture of human islets that developed *ex vivo* in the *scid* mice is similar to that of normal adult human islets.

During pancreatic development in rodents, beta cells are derived from Pdx1-positive progenitors with a high proliferative potential [6], [7]. When the cells enter the endocrine pathway and start to express the transcription factor Neurogenin3, their proliferation rate decreases sharply [32], [33]. During the later perinatal stage, beta cells derived from Neurogenin3 progenitors and located within the islets reenter the cell cycle [34], [35]. Thus, the number of insulin-positive cells present in a rodent islet is probably dependent on the proliferation rates of both progenitor and mature beta cells. We reasoned that if the beta cells present in a single human islet originate from a unique progenitor, Pdx1-positive progenitor cells and/or mature beta cells should proliferate. We found that a large number of Pdx1-positive progenitor cells stained positive for Ki67 10 days after transplantation, demonstrating the proliferative potential of such cells. We also found that some of the beta cells present in human islets that developed in *scid* mice were proliferative. The low rate of beta-cell proliferation is consistent with beta-cell proliferation rates found in prenatal human pancreas development [22]. Thus, with such data, we could not exclude the possibility that beta cells present in a single islet may originate from a unique progenitor that proliferates at the progenitor stage and/or at the beta-cell stage.

Here, we used low multiplicity of infection to transduce human fetal pancreases with lentiviruses expressing GFP under the control of the rat insulin II promoter. This promoter has previously been used for lentivirus-mediated transgene expression restricted to rodent beta cells [15]. Our findings described here demonstrate that restricted expression is also obtained in mature human beta cells. We generated heterogeneous islets composed of some beta cells positive and others negative for the transgene product by transducing a limited number of human fetal pancreatic progenitors and consequently conclude that human islets derive from more than one progenitor.

Little is known about tissue morphogenesis in humans mainly due to the absence of informative experimental models. In this study, we developed a unique model combining *ex vivo* organogenesis from human fetal tissue and cell type-specific lentivirus-mediated gene transfer. This approach resulted in the generation of human transgenic-like developing tissue. This original model allowed a dynamic analysis of human islet formation during development. It can now be generalized as a powerful transgenic-like tool to examine in a dynamic fashion the genetic processes involved in tissue formation in humans.

## Materials and Methods

### Human tissues

We extracted human fetal pancreases from tissue fragments obtained immediately after elective termination of pregnancy. Terminations were performed by aspiration between 9 and 11 weeks of development, in compliance with French legislation and the guidelines of our institution, as previously described [16], [20]. Ethics approval was obtained from the Agence de Biomedecine, the French competent authority along with maternal written consent. Warm ischemia lasted less than 30 minutes. Gestational ages were determined on the basis of time since the last menstrual period, crown-rump length measured by ultrasonography, and hand and foot morphology.

Crude human primary islet cell preparations were isolated from one human donor organ (from a 16-year-old girl) and cultured as described previously [36]. The organs were procured by a European hospital affiliated with the Eurotransplant Foundation (Leiden, the Netherlands) and processed by the Beta-Cell Bank of the Juvenile Diabetes Research Foundation Center for Beta-Cell Therapy in Diabetes. Full written consent was given by the parents to use the donor organ for transplantation and research purposes according to the Belgian laws. Ethics approval to use endocrine enriched cells derived from donor organ was given to Dr. Harry Heimberg by the Medical ethical committee of the University Hospital of the Vrije Universiteit Brussel. Collagenase digests were separated by Ficoll gradient purification into an islet-enriched fraction and an exocrine fraction. The islet-enriched fraction was cultured for three days [36]. On the day of viral infection, the islet cell preparation contained 15% insulin-positive cells and 20% cytokeratin 19-positive duct cells.

### DNA constructs and recombinant lentivirus production

The vectors, pTRIP ΔU3.CMV-eGFP [37] and pTRIP ΔU3.RIP405-eGFP [15], express the eGFP reporter gene under the control of an internal CMV promoter and a 405 bp fragment of the rat insulin II promoter (RIP405), respectively.

Lentiviral vector stocks were produced by transient transfection of 293T cells with the p8.7 encapsidation plasmid (ΔVprΔVifΔVpuΔNef) [38], the VSV glycoprotein-G-encoding pHCMV-G plasmid [39], and the pTRIP ΔU3. recombinant vector as previously described [40]. Supernatants were treated with DNAse I (Roche Diagnostic) prior to ultracentrifugation and the resulting pellet was resuspended in Phosphate Buffered Saline, separated into aliquots and frozen at −80°C until use. The amount of p24 capsid protein was quantified by the HIV-1 p24 ELISA antigen assay (Beckman Coulter). The transduction efficiency of each vector stock was determined as previously described by infecting Min6 cells [15].

### Lentivirus-mediated gene transfer in human fetal pancreases and islet cell preparation

Recombinant lentiviruses were used to transduce partially dissociated human fetal pancreatic tissue and the adult human islet-enriched fraction. Human fetal tissue was incubated with 2.10^5^ TU of either CMV or RIP vectors for two hours at 37°C with 5% CO_2_ atmosphere, in 200 µl of RPMI 1640 medium supplemented with 10% heat-inactivated fetal calf serum, HEPES (10 mM), L-glutamine (2 mM), non essential amino acid (Invitrogen), penicillin (100 units/ml)-streptomycin (100 µg/ml) and 10 µg/ml DEAE-dextran. Tissues were then washed twice with HEPES Buffered Saline Solution (HBSS, Invitrogen) and kept on ice until transplantation into *scid* mice. Eight fetal human pancreases were used in this study. Two were transduced with the pTRIP ΔU3.CMV-eGFP and analyzed 10 days after transplantation. Six were transduced with the pTRIP ΔU3.RIP405-eGFP. The first 3 were analyzed 10 days after transplantation and the remaining three 4.5 months after transplantation.

Adult human islet cells were transduced with 4.10^6^ TU of pTRIP ΔU3.RIP405-eGFP lentiviral vector for 48 h at 37°C in Ham F10 serum-free medium. Following infection, cells were washed and cultured for a further 24 h before analysis.

### Transplantation of infected human fetal pancreases

Male *scid* mice (Charles River Laboratories, L'arbresle, France) were maintained in isolators.

Using a dissecting microscope, pancreases were implanted under the kidney capsule as previously described [15], [16], [20]. Briefly, the left kidney was exteriorized; a small transverse incision was made through the capsule on the ventral surface of the kidney, near the inferior pole. A small silicon cylinder was pushed under the capsule to provide a sealed space to confine the transplanted cells and tissues [41]. The tissues were then introduced into the cylinder using forceps and/or a Hamilton syringe. Ten and 135 days after transplantation, mice were sacrificed, the kidney removed, and the graft dissected. Tissue was fixed with 3.7% formaldehyde prior to embedding in paraffin for immunohistological analysis.

### Immunochemistry

For human fetal pancreases, 4 µm-thick sections were cut on gelatinized glass slides, deparaffinized in toluene, and rehydrated. Immunofluorescence staining was performed as previously described [32], [42]. The primary antibodies were: rabbit anti-human insulin (DiaSorin, Antony, France 1/500), mouse anti-human insulin (Sigma, Saint-Quentin Fallavier, France 1/1000), rabbit anti-glucagon (DiaSorin, 1/1000), mouse anti-Ki67 (Dako, Trappes, France 1/50), mouse anti-GFP (Euromedex, Souffelweyersheim, France, 1/200) and rabbit anti-human PDX1 (1/2000 [20]). Secondary antibodies were fluorescein anti-mouse and Texas-red anti-rabbit and anti-mouse (Jackson immunoresearch, Monluçon, France 1/200), and AlexaFluor 488 anti-rabbit (Molecular Probes, Cergy Pontoise, France 1/200).

The suspension cultures of transduced islet cells were fixed for 0.5 h in 4% paraformaldehyde and pelleted in 2% agarose before paraffin embedding. Immunohistochemical analysis was performed on paraffin sections using guinea pig polyclonal anti-insulin (1/3000, generated at the Diabetes Research Center, Brussels), rabbit polyclonal anti-amylase (1/500, Sigma), goat polyclonal anti-GFP (1/100, Abcam, Cambridge, UK) and mouse monoclonal anti-cytokeratin 19 (1/500, Dako). Before incubation with the first antibody, sections for CK19 staining were treated with trypsin for antigen retrieval. Secondary antibodies were all from Jackson ImmunoResearch Laboratories: Cy3-labeled anti-rabbit (1/800), anti-mouse (1/400), anti-guinea pig (1/400), and FITC-labeled anti-goat (1/200).
